# Facile Synthesis of Hydrogen-Substituted Graphdiyne Powder via Dehalogenative Homocoupling Reaction

**DOI:** 10.3390/nano13061018

**Published:** 2023-03-11

**Authors:** Jiayi Yin, Jizhe Liang, Chunxue Yuan, Wei Zheng

**Affiliations:** College of Materials Science and Engineering, Tongji University, Shanghai 201804, China

**Keywords:** carbon material, graphdiyne, hydrogen-substituted graphdiyne, dehalogenative homocoupling reaction

## Abstract

Graphdiyne and its analogs are a series of artificial two-dimensional nanomaterials with sp hybridized carbon atoms, which can be viewed as the insertion of two acetylenic units between adjacent aromatic rings, evenly expanded on a flat surface. Although developed in recent years, new synthetic strategies for graphdiyne analogs are still required. This work proposed a new method to prepare hydrogen-substituted graphdiyne powder via a dehalogenative homocoupling reaction. The polymerization was unanticipated while the initial goal was to synthesize a γ-graphyne analog via Sonogashira cross-coupling reaction. Compared with previous synthetic strategies, the reaction time was conspicuously shortened and the Pd catalyst was inessential. The powder obtained exhibited a porous structure and high electrocatalytic activity in the hydrogen/oxygen evolution reaction, which has the potential for application in electrochemical catalysis. The reported methodology provides an efficient synthetic strategy for large-scale preparation.

## 1. Introduction

In recent years, carbon-rich materials have been exploited and have attracted much research for their prosperity and potential application. New carbon allotropes have been experimentally realized, such as single-walled carbon nanotubes, few-layer graphene, schwarzites, and graphyne [1,2,3,4]. Graphyne and its analogs are a series of two-dimensional nanomaterials containing sp and sp^2^ hybridized carbon atoms [5]. As a new member of the carbon family, graphyne is endowed with unique physical and chemical properties by sp and sp^2^ hybridized carbon atoms, which makes a considerable difference from graphene. γ-graphyne can be viewed as the insertion of some acetylenic units between adjacent aromatic rings evenly expanded on a flat surface, dictating a horizontal structure with periodic dispersed pores. γ-graphyne is named after the number of acetylenic bonds between two adjacent aromatic rings, in the manner of graphdiyne, graphtriyne, and so on [6]. The aromatic rings and acetylenic bonds compose an extended π-conjugated system, rendering the moderate band gap and high charge carrier mobility of single-layer γ-graphyne [7]. Combining electronic properties and nanoscale porosity, γ-graphyne shows enormous potential in gas separation, catalysis, water purification, energy storage, supercapacitors [8,9,10,11,12,13,14,15,16,17], etc.

In 2010, Li and his co-workers pioneered synthesized graphdiyne film on the surface of copper foil via an in situ cross-coupling method using hexaethynylbenzene as a precursor [18]. Since then, the synthetic strategies for graphdiyne and its analogs (GDYs) have advanced rapidly and have been divided into wet chemistry and dry chemistry. The homocoupling reaction of monomers with terminal alkynyl groups, such as Glaser, Glaser-Hay, Eglinton, and Sonogashira coupling reactions are the keys of wet chemistry and have been wildly exploited to prepare new types of GDYs [10,19,20,21,22,23,24,25,26,27,28]. These reactions frequently require the high reactivity of monomers, which increases the risk of oxidative deterioration during the synthetic process. To retard the oxidative deterioration in preparation, trimethylsilyl is introduced, which enhances the stability of monomers as a protection group [29,30]. However, trimethylsilyl generates various by-products and significantly reduces efficiency. The more efficient and stable precursors are worthy of research. Apart from wet chemistry, dry chemistry is a formidable strategy for the accurate synthesis of molecular structures, such as chemical vapor deposition and surface chemistry. In 2015, we designed 1,3,5-tris(bromoethynyl)benzene (tBEP) and successfully constructed a two-dimensional molecular network with acetylenic scaffoldings on Au(111) under an ultrahigh vacuum [31]. We then prepared free-standing hydrogen-substituted graphdiyne (HsGDY) films in solution via the dehalogenative homocoupling reaction of terminal alkynyl bromide molecules [32]. The terminal alkynyl bromides of tBEP imparted higher stability than terminal alkynyl and fewer by-products than trimethylsilyl. Furthermore, this revealed higher selectivity, which shortened the time of the synthetic process and reduced the need for extra heating.

**Figure 1:**
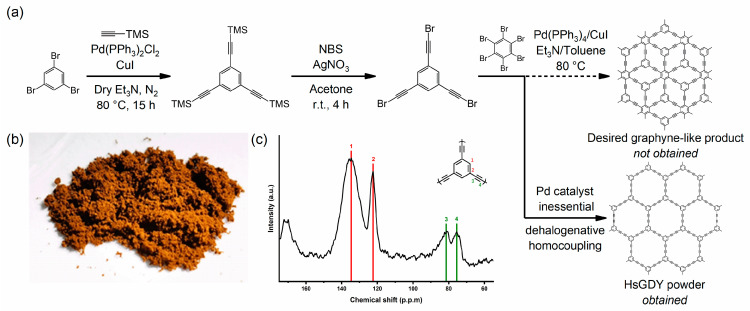
(**a**) Synthesis of HsGDY powder. (**b**) A photograph of HsGDY powder. (**c**) The ^13^C solid-state NMR spectrum of HsGDY powder.

In this work, our initial goal was to synthesize a γ-graphyne analog with terminal alkyne bromides via a dehalogenative coupling reaction between tBEP and hexabromobenzene, using a Sonogashira cross-coupling reaction for terminal alkynes bromides and terminal bromides (Figure 1a). A yellow-brown powder and a small number of black powders as side products were obtained after preparation. The powders were found to be insoluble in organic solvents and were stable under strong acid and alkali conditions, such as 4 M aqueous HCl, 1 M aqueous H_2_SO_4_, and 4 M aqueous NaOH. The powder was analyzed by ^13^C solid-state Nuclear Magnetic Resonance (NMR), X-ray Photoelectron Spectroscopy (XPS), Fourier Transform-InfraRed spectroscopy (FT-IR), Raman spectroscopy, X-ray Powder Diffraction (XRD), and some chemical functional tests, revealing that sp and sp^2^-hybridized carbons were present, which could be a graphyne analog. Nevertheless, the analysis results indicated that the yellow-brown powder should be a porous carbon material with sp and sp^2^ hybridized carbon atoms and a two-dimensional structure, which had properties and characteristics that were very close to the theory of HsGDY (Figure 1b). We believe the product obtained can be referred to as HsGDY powder instead of the desired graphyne-like material, while the black powder should be a conjugated porous carbon material (CPCM). We attributed this to the dehalogenative homocoupling reaction of tBEP, which had higher reactivity in the homocoupling reaction rather than the cross-coupling reaction. The reaction selectivity of tBEP indicates that it is an efficient precursor for the rapid and bulk preparation of graphyne-like materials. The HsGDY powder, synthesized through a dehalogenative homocoupling reaction using tBEP as a precursor, showed good electronic properties and can be applied in electrochemical catalysis.

## 2. Materials and Methods

### 2.1. Chemicals and Components

1,3,5-Tribromobenzene (98.0%, C_6_H_3_Br_3_, Adamas, Shanghai, China), Bis(triphenylphosphine)palladium chloride (98.0%, PdCl_2_(PPh_3_)_2_, Adamas, Shanghai, China), Copper(I) iodide (99.0%, CuI, Adamas, Shanghai, China), Trimethylsilylacetylene (98.0%, (CH_3_)_3_SiC≡CH, Adamas, Shanghai, China), N-Bromosuccinimide (98.0%, NBS, Greagent, Shanghai, China), Triethylamine (99.0%+, Et_3_N, Greagent, Shanghai, China), Hexane (99.0%+, C_6_H_14_, Greagent, Shanghai, China), Acetone (99.5%, (CH_3_)_2_CO, Sinopharm, Beijing, China), Silver nitrate (99.0%, AgNO_3_, Sinopharm, Beijing, China), Toluene (99.5%, PhMe, Sinopharm, Beijing, China), Dichloromethane (99.5%, CH_2_Cl_2_, Greagent, Shanghai, China), Absolute ethanol (99.0%, EtOH, Greagent, Shanghai, China), and Ammonia solution (29%, NH_3_(aq), Adamas, Shanghai, China).

### 2.2. Synthesis of tBEP Polymer

#### 2.2.1. 1,3,5-Tris((trimethylsilyl)ethynyl)benzene

To a mixture of 1,3,5-tribromobenzene (1.26 g, 4 mmol), PdCl_2_(PPh_3_)_2_ (0.45 g, 0.64 mmol) and CuI (0.22 g, 1.2 mmol) in dry Et_3_N (30 mL) was added trimethylsilyacetylene (2.0 mL, 14.4 mmol). The reaction mixture was stirred at 80 °C for 15 h under N_2_ and monitored by thin-layer chromatography. After solvent removal, the residue was purified by column chromatography on silica gel (eluent: n-hexane, R_f_ = 0.4) to afford 1,3,5-tris((trimethylsilyl)ethynyl)benzene (0.9320 g, 2.55 mmol, 64% yield) as a white solid.

#### 2.2.2. 1,3,5-Tris(bromoethynyl)benzene (tBEP)

To a solution of 1,3,5-tris((trimethylsilyl)ethynyl)benzene (0.367 g, 1.00 mmol) in acetone (20 mL) was added NBS (0.641 g, 3.60 mmol) and AgNO_3_ (0.102 g, 0.60 mmol, 60 mmol%) at r.t. After 4 h (monitored by thin-layer chromatography), the reaction mixture was diluted with n-hexane and the crystals formed were filtered off. The filtrate was concentrated under reduced pressure and purified by column chromatography on silica gel (eluent: n-hexane, R_f_ = 0.7) to afford tBEP (0.371 g, 0.96 mmol, 96% yield).

### 2.3. Synthesis of HsGDY Powder

To a solution of tBEP (0.160 g, 0.41 mmol) in Et_3_N (6 mL) and Toluene (6 mL) was added CuI (0.01 g, 0.05 mmol). The reaction mixture was stirred at 80 °C for 30 min under N_2_. A solid product was formed, centrifuged out, and subsequently washed with dichloromethane, ethanol, acetone, ammonia solution, and acetone several times to remove any unreacted monomers or oligomers. After solvent removal, the product was dried at 100 °C for 24 h to afford HsGDY powder as a yellow-brown solid and a very small amount of CPCM as a black solid.

## 3. Results and Discussion

The HsGDY powder was synthesized unexpectedly while our initial desired product was a new γ-graphyne analog. The reaction proceeded in a 50 mL 3-neck flask with a 4 mm × 10 mm stirring bar. After the reaction had completely stopped, the brownish-yellow powder was dispersed on the walls of the flask and a small portion was dispersed in the solution, while some black powder was distributed at the bottom of the flask and quite tightly stuck to the wall. The yellow-brown powder was easily carried out of the flask by organic reagent and dried; this was referred to as HsGDY powder. The black powder that was stuck to the bottom of the flask was scraped off and dried; this was referred to as CPCM. The obtained CPCM was much less than HsGDY powder in the reaction. The reactivity of tBEP was higher than expected, so homocoupling occurred to form a self-assembled structure instead of heterocoupling with hexabromobenzene. We experi fluent mented with the condition that affected the reaction (Table S1). The experiment indicated that the dehalogenative homocoupling reaction of tBEP can proceed under the necessary conditions of Toluene solvent, Cu(I) catalyst, heating, and some Lewis bases, such as Pyridine and Et_3_N. The Pd catalyst failed to promote the desired reaction and was inessential in the dehalogenative homocoupling reaction. The reaction proceeded very fast and completed in dozens of minutes. We previously prepared the HsGDY film under Pyridine/NaOH(a.q.) and copper foil at room temperature [32]. The use of Cu(I) catalyst instead of copper foil to reproduce the preparation with a view to obtain some product but nothing. We speculate that the growth substrate had a different manner to affect film formation than we considered. A very small amount of CPCM was also obtained in the experiments when heated overtime and used no Cu(I) catalyst.

The ^13^C solid-state NMR spectrum of HsGDY powder revealed that there were signals of four types of carbon atoms; see Figure 1c. The peaks at 135.0, 122.5, 82.0 and 75.1 ppm corresponded to aromatic C−C, aromatic C−H, C(sp)−C(sp^2^), and C(sp)−C(sp), respectively [26]. The main peaks were not sharp and the secondary peaks were widely distributed, indicating that the skeletal structure of the HsGDY powder was not intact and had suffered some breakage.

**Figure 2:**
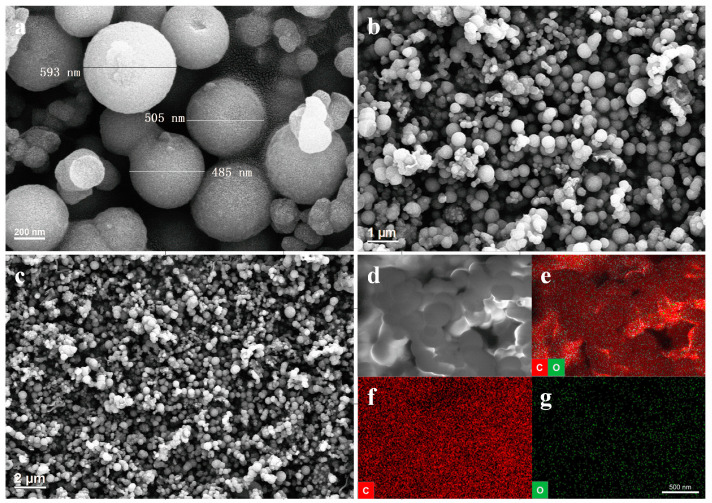
(**a**–**c**) SEM images of HsGY Powder. (**d**–**g**) EDS mapping images of C + O (**e**), C (**f**), and O (**g**) of HsGY Powder.

The morphology details of HsGDY powder were studied using scanning electron microscopy (SEM). The SEM images of HsGDY under different degrees of magnification are shown in Figure 2. The surface morphology of HsGDY powder presented a structure of random accumulation that was constructed by spherical particles. The diameter of spherical particles ranged from 350 nm to 700 nm, as shown in Figure 2a. The particles had an uneven surface and the smaller size particles appeared amorphous and no longer remained spherical. It can be interpreted that the structure was formed without substrates, rendering the phenomenon of curling and agglomeration of the sample material with a two-dimensional structure. The structure of the CPCM was similar to HsGDY powder, as shown in Figure S1.

The energy dispersive spectrometer (EDS) was employed to analyze the composition of element of HsGDY powder. Theoretically, only two elements (carbon and hydrogen) were present in the HsGDY powder, while the carbon atoms included only two kinds of hybridized atoms (sp and sp^2^) [33,34]. Thus, an energy dispersive spectrometer (EDS) was employed to analyze the elemental composition of the samples. The powders were mainly composed of carbon and oxygen, mostly carbon, as depicted in Figure 2e. The carbon was scattered throughout the sample at a very high density, while the oxygen was much less. Deposition of oxygen in the air and unavoidable defects were responsible for the presence of oxygen. The EDS results showed that the relevant content of carbon in powder was 93.56%, verifying HsGDY powder as a carbon-rich material (Figure S7).

**Figure 3:**
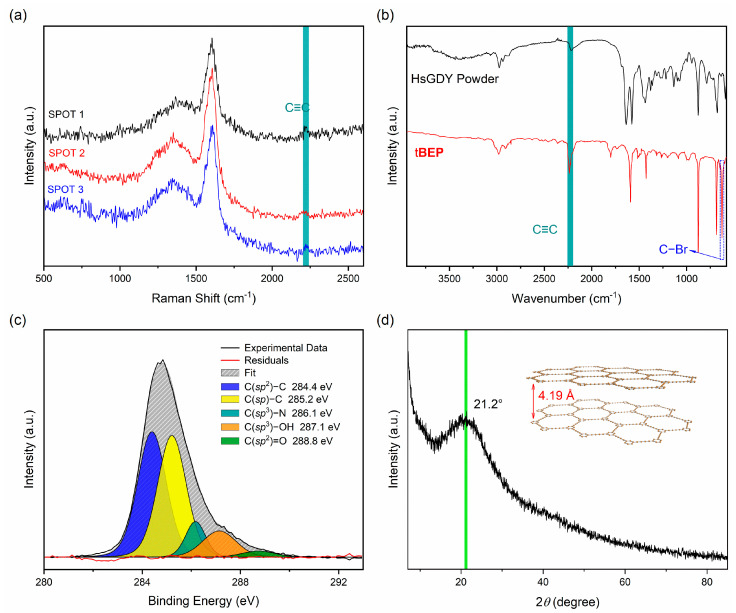
The structure of the HsGDY powder. (**a**) Raman spectra of random spots on the surface. (**b**) FT-IR spectra of the powder and tBEP. (**c**) XPS high-resolution C 1s spectrum of the powder. (**d**) XRD patterns of the powder.

Raman spectroscopy has proven to be an effective tool in characterizing the structure of carbon materials. It is sensitive to the symmetrical vibration of skeleton and functional groups, while FT-IR shows sensitivity to the asymmetrical vibration of functional groups. Both approaches are usually combined to get a full vision of the analysis of a product [35,36,37]. In the characterization of GDYs, Raman spectroscopy and FT-IR can be used to detect the bonding structure of carbon. As depicted in Figure 3a, two major peaks were found in the Raman spectra of the HsGDY powder. The D-band located at 1352 cm^−1^ was assigned to the defects or edges, and the G-band located at 1593 cm^−1^ indicated the in-plane stretching vibration of the sp^2^ hybridized carbon atoms. There was a weaker peak at 2213 cm^−1^ that should correspond to the vibration of alkynyl bonds [33]. We noted that the peaks of the vibration of alkynyl bonds were generally weak, according to previous reports, including films grown using copper sheets as substrates [25,26,29]. This suggests that the structures of the graphyne-like materials synthesized through wet chemical methods were generally not very good. As depicted in Figure 3b, several obvious peaks were found in the FT-IR spectra. The peak at 1590 cm^−1^ was generated by the vibration of the benzene ring skeleton, and the peaks at 2963 cm^−1^ and 876 cm^−1^ were assigned to the different vibration modes of C−H bonds in the aromatic rings, indicating the existence of benzene rings in the samples. The peak at 2188 cm^−1^ was generated by the typical stretching vibration of the alkynyl bond, which had a conspicuous peak compared with the Raman spectra. The signal peak was consistent with the Raman spectra, indicating the presence of an alkynyl bond structure [16,38]. We also compared the Raman and FT-IR spectra of the tBEP monomer, CPCM, and HsGDY film to the powder (Figures S2 and S3), where there conspicuous intensity upswing of the peak at 994 cm^−1^ was observed in the Raman spectrum and at 687 cm^−1^ in the FT-IR spectra. These two signal peaks belonged to the stretching vibration of the C−Br bonds, which significantly decreased after the reaction. In combination with the spectra mentioned, the diversities of these two signal peaks demonstrated the occurrence of the dehalogenative homocoupling reaction and the formation of the HsGDY powder. Figure 3d shows the XRD patterns of the HsGDY powder. The peak at 21.2° corresponded to 4.19 Å as an interlayer distance, which was compatible with HsGDY films previously reported [26,39]. The XRD analysis indicated that the HsGDY powder we obtained was amorphous owing to the conformational fluctuation of the structure and the arduousness of accurately modulating the structure formed by the wet chemistry approach.

To identify the hybridization status and relative content of the carbon, X-ray photoelectron spectroscopy (XPS) was conducted. HsGDY powder is mainly composed of carbon elements (82.82%) (Figure S4). The presence of oxygen elements (11.21%) was attributed to the oxidation of alkynyl groups, at the edges mainly, and some adsorption in the air secondarily, which is consistent with EDS elemental mapping. The presence of nitrogen elements (6.57%) at the 399.8 eV position was owing to the Et_3_N used in the reaction and ammonia water used in product washing. The C 1s peak was deconvoluted into five subpeaks: 284.4 eV, 285.2 eV, 286.1 eV, 287.1 eV, and 288.8 eV. The peaks at 284.4 eV and 285.2 eV applied to C(sp^2^)−C and C(sp)−C, respectively, and the area of C(sp^2^) and C(sp) was nearly equal, which was consistent with the theoretical structure of HsGDY powder [28,33,34]. The peak at 286.1 eV referred to C(sp^3^)-N for the remaining nitrogen in the powder. The peaks at 287.1 eV and 288.8 eV applied to hydroxy (C−OH) and carbonyl (C=O), respectively, and the O 1s peak was deconvoluted in the same rule [40]. The CPCM had a similar component to the HsGDY powder with less oxygen and nitrogen doped-in material (Figure S6). The appearance of the carbon-oxygen bonds and carbon-nitrogen bonds indicated that the HsGDY powder was an oxygen-doped and nitrogen-doped material, which partly altered the electronic structures and may eventually affect the properties.

**Figure 4:**
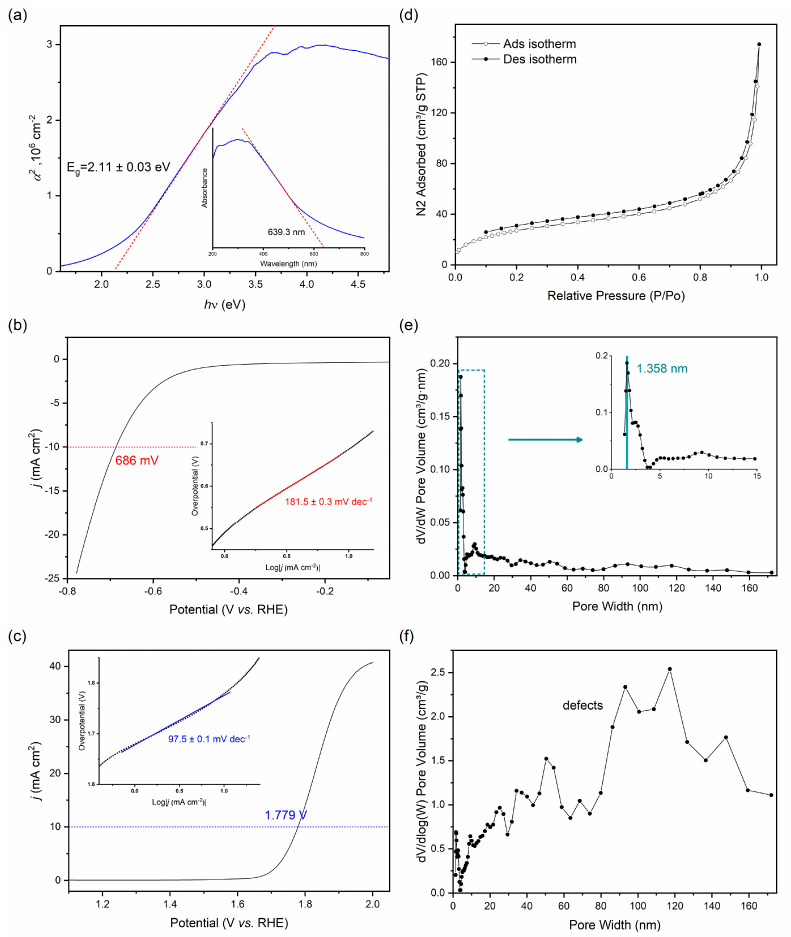
(**a**) Determination of the optical band gap from UV-visible spectrophotometry of the HsGDY powder. (**b**,**c**) HER (**b**) and OER (**c**) catalytic performance of the HsGDY powder. (**d**) Nitrogen adsorption-desorption isotherm. (**e**,**f**) Pore width distribution (**e**) and surface area (**f**) for the HsGDY powder, calculated after fitting a DFT model to the adsorption data.

The ultraviolet-visible spectrophotometry of the HsGDY powder showed a broad absorption band between 350 to 650 nm, and the sample had a clear cut-off wavelength of around 640 nm (Figure 4a). The absorbance of photon energy within the electronic edge depended on the electronic band gap, E_g_, and determined the type of semiconductor. The spectrum of the sample could convert to a Tauc plot to estimate the electronic band gap [41]. In the Tauc formula, (α*h*υ)^1/n^ = A(*h*υ − E_g_), the absorption coefficient α is proportional to the absorbance of photon energy and A is a constant. Within the classical theory of optical absorption of crystalline direct band gap semiconductors, n is expected to be 1/2 for direct bandgap materials and to be 2 for indirect bandgap materials, correspondingly. For most γ-graphyne analogs, it has a direct band gap [42]. Therefore, the Tauc plot can be shown in terms of α^2^ versus *h*υ and achieve a curve with a linear region. Extrapolating the linear region of the curve to the abscissa yields the energy, and E_g_ can be estimated for a value of 2.11 ± 0.03 eV. In contrast to the HsGDY film we previously reported (Figure S8), the powder had a wider absorption band and smaller electronic band gap, which demonstrated better electronic properties of the powder [32].

Furthermore, the HsGDY powder offered extensive tunnels and vast interspaces for ion transport and storage due to the extended π-conjugated system and the two-dimensional porous structure [17,26,43]. The electrocatalytic performance of the powder was investigated with typical three-electrode measurements at room temperature for hydrogen evolution reaction (HER) and oxygen evolution reaction (OER). The as-prepared HsGDY powder was applied with Nafion/isopropyl on the surface of carbon paper and used as the working electrode, with a Pt counter electrode and a Hg/HgO (in 1 M KOH) reference electrode at a scan rate of 5 mV/s. The overpotential of HER required for the HsGDY powder to reach the current density (*j*) of 10 mA cm^−2^ was 685 mV with a Tafel slope of 181.5 mV dec^−1^, as shown in Figure 4b. OER required an overpotential of 1.779 eV with a Tafel slope of 97.5 mV dec^−1^ to achieve the current density (*j*) of 10 mA cm^−2^ (Figure 4c). The HsGDY powder exhibited an exceptional HER/OER electrocatalytic activity in comparison with the HsGDY film we previously obtained [32,44]. However, the existence of oxygen in the powder may have reduced the continuity of the π-conjugated system, which could have significantly affected the electrical conductivity and the charge carrier mobility [45]. The effect of doped-in nitrogen was uncertain because of the difficulty of valuing its contribution to the structure. We speculated that the difference in electrocatalytic performance between the powder and the film was caused by the curling of the two-dimensional structure, which means that the powder had a larger surface area and interspace in the same size of electrode surface. The electrocatalytic performance of the HsGDY powder should be improved when other metal atoms are doped in, since the sp hybridized linkages can directly remain in the active site and chelate metal atoms to form a complex catalytic system [14,17,44].

The porosity of the HsGDY was investigated by collecting the nitrogen gas adsorption and desorption isotherms at 77.3 K, as shown in Figure 4d. The powder generated Type I nitrogen gas sorption isotherms with H3 hysteresis loops, according to the IUPAC classification. The nitrogen adsorption of the powder showed a very sharp uptake in the beginning and a slow rise with the pressures increased, which is a significant feature of microporous material [46]. As the pressures continued to increase, the adsorption isotherm rose sharply again and was not completely fitted with the desorption isotherm, which is a typical feature of non-rigid aggregates of lamellar particles with the network of macropores. The network of macropores was a consequence of structural defects and was not fully filled with pore condensation. The Brunauer–Emmett–Teller (BET) model and t-method were not applied to estimate the apparent surface area of microporous materials [47]. The pore width distribution was calculated after fitting the destiny function theory (DFT) model (Figure 4e,f), which gave a specific surface area of 5.621 m^2^ g^−1^ and a main pore size of 1.358 nm. The specific surface area for the sample was unusually small compared to the graphyne-like materials reported previously, which normally have a specific surface area of >300 m^2^ g^−1^ [26]. On the other hand, the main pore size for the sample was smaller than the theoretical pore size of single-layer HsGDY (1.63 nm), indicating the stack of the HsGDY layer in the framework. The pore size was mainly concentrated around the size of the micropore, while the mesopores and macropores give rise to the minority in distribution but the majority in the surface area. We attributed these to the impaired structure of the sample, which only formed the periodic two-dimensional multilayer structure on a small-space scale. On the larger-space scale, the two-dimensional framework of the sample turned out to be irregular and twisted, eventually rendering a huge number of defects. In contrast to the HsGDY film, the powder was imperfect in nitrogen gas adsorption. Besides the issues we analyzed, the fabrication of powder was based on curling and aggregation in the polymerization, which turned out to be barriers to subsequent assembly and led to irregularities in the structure.

## 4. Conclusions

In summary, we utilized tBEP as the monomer and prepared HsGDY powder via a dehalogenative homocoupling reaction. Compared with previous methods, our reaction proceeded without a Pd catalyst, and the reaction time was conspicuously shortened to dozens of minutes, endowing the potential for industrial preparation on a large scale. The terminal alkynyl bromide functional groups combined high stability with high reactivity, showing great potential in the synthesis of graphyne analogs. HsGDY powder is a free-standing, carbon-rich material with an uneven surface and porous structure. Qualitative analysis results demonstrated a structure with generous aromatic rings and alkyne bonds, substantiating the successful synthesis of a graphdiyne-like nanomaterial. Quantitative measurements demonstrated good performance in semiconductor and electrochemical activity, better than the films we previously obtained, but with poor performance in gas adsorption. The powder still had many defects and the preparation method can be further improved. For the preparation, optimal reaction conditions still need to be tried. The doped-in oxygen atoms and nitrogen atoms should be decreased during the reaction. The characteristics and synthesis mechanism of the by-product CPCM have not been investigated clearly. With improved experimental methods, it may be possible to obtain a large number of CPCMs for further testing to investigate their performance. For the product, the characteristics and properties of the powder need to be improved. The major problem was obtaining a higher quality product with a crystal structure, which was also the key challenge to developing the synthesis of graphyne-like materials. The powder form has certain disadvantages in the preparation of crystalline samples for the lack of substrate, while the film form has advantages in fabricating an even structure. However, powders can be prepared without a substrate, making them conducive to large-scale preparation. The future applications of the powder could also be more extensively researched. The improved powder may have potential in the fields of water purification, energy storage, supercapacitors, and so on. This work proposed an efficient method to synthesize HsGDY as a potential solution for industrial preparation and showed the special performance of the powder in electrocatalytic activity.

## Data Availability

The data presented in this study are available on request from the corresponding author.

## Supplementary Materials

The following supporting information can be downloaded at: https://www.mdpi.com/article/10.3390/nano13061018/s1. Table S1: Experiment of the reaction condition to obtain HsGDY powder. The reactant only contains tBEP, with a heating temperature up to 80 °C, and the reaction takes longer at lower heat temperatures. All experiments were performed under a nitrogen atmosphere. Figure S1: The photograph (a) and SEM images (b,c) of CPCM. Figure S2: (a) Raman spectrum of tBEP monomer. (b) Raman spectrum of CPCM and HsGDY film. Figure S3: FT-IR spectrum of CPCM and tBEP. Figure S4: XPS total spectrum of HsGDY powder. Figure S5: XPS high resolution of O 1s spectrum of HsGDY powder. Figure S6: XPS high resolution of (a) C 1s and (b) O 1s spectrum of CPCM. Figure S7: EDS analysis of HsGDY powder. Figure S8: (a) The UV-visible spectrophotometry of HsGDY film (b) Determination of optical band gap of HsGDY film from the Tauc plot.
