# Supplementary material for: Facile Synthesis of Hydrogen-Substituted Graphdiyne Powder via Dehalogenative Homocoupling Reaction
Source: Nanomaterials (Basel). 2023 Mar 11;13(6):1018. doi: 10.3390/nano13061018 (PMC10055811; doi:10.3390/nano13061018)
Supplement: Supplementary file 1 [file nanomaterials-13-01018-s001.zip › nanomaterials-2244927-supplementary.pdf]

Supporting Information

Facile Synthesis of Hydrogen-Substituted Graphdiyne Powder via Dehalogenative Homocoupling Reaction

Jiayi Yin, Jizhe Liang, Chunxue Yuan and Wei Zheng *

College of Materials Science and Engineering, Tongji University, Shanghai 201804, China
* Correspondence: zhengwei@tongji.edu.cn

Catalyst	Lewis bases	Condition	Product obtained or not
CuI	Et_3N	Toluene/heating	Y
CuI	Pyridine	Toluene/heating	Y
CuI	DMSO	Toluene/heating	Y
CuI	THF	Toluene/heating	N
CuI	DMF	Toluene/heating	Y
CuI	DMAC	Toluene/heating	N
CuI	Et_3N	Toluene	N
CuI	Pyridine	Toluene	N
CuI	Pyridine	NaOH(a.q.)	N
None	Et_3N	Toluene/heating overtime	A few black powder (CPCM)
None	Pyridine	Toluene/heating overtime	A few black powder (CPCM)
CuI	None	Toluene/heating	N

Table S1. Experiment of the reaction condition to obtain HsGDY powder. The Reactant only contains tBEP, heating temperature up to 80 °C and reaction takes longer at lower heating temperature. All experiment is performed under nitrogen atmosphere.

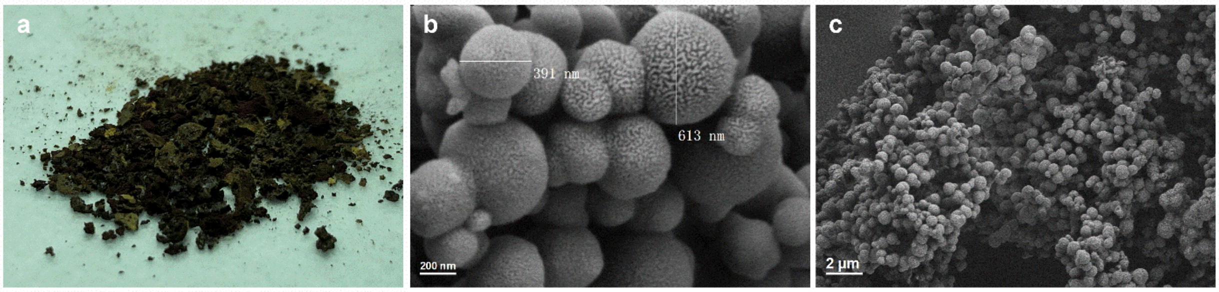

Figure S1. The photograph (a) and SEM images (b,c) of CPCM

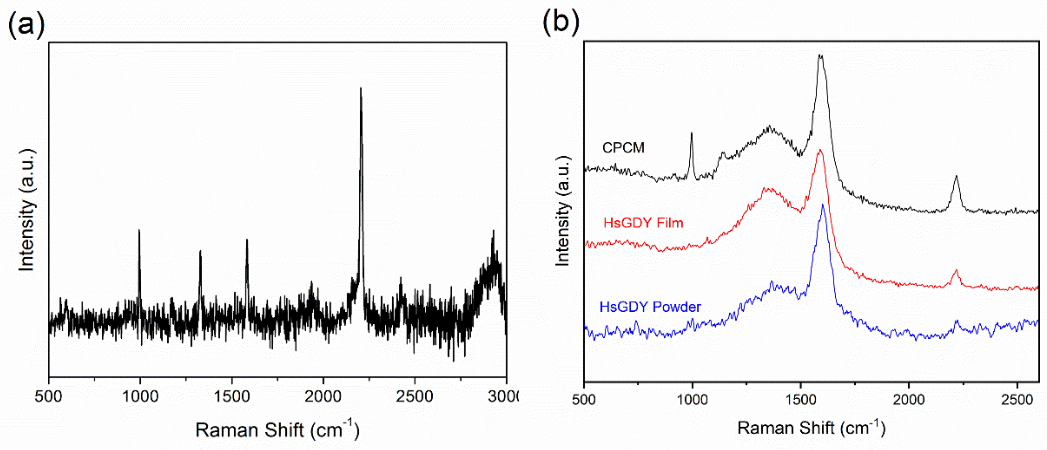

Figure S2. (a) Raman spectrum of tBEP monomer. (b) Raman spectrum of CPCM and HsGDY film.

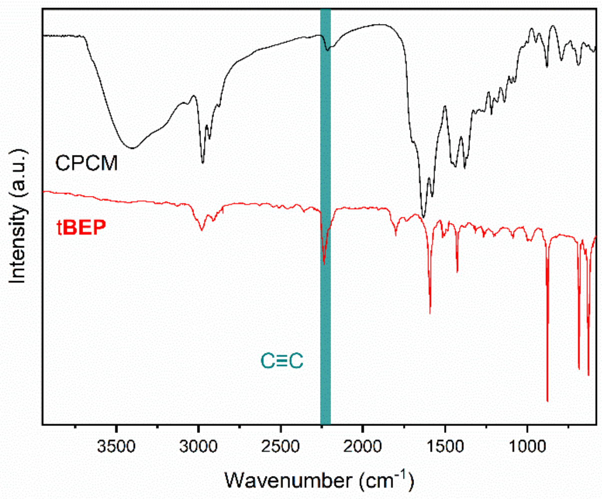

Figure S3. FT-IR spectrum of CPCM and tBEP.

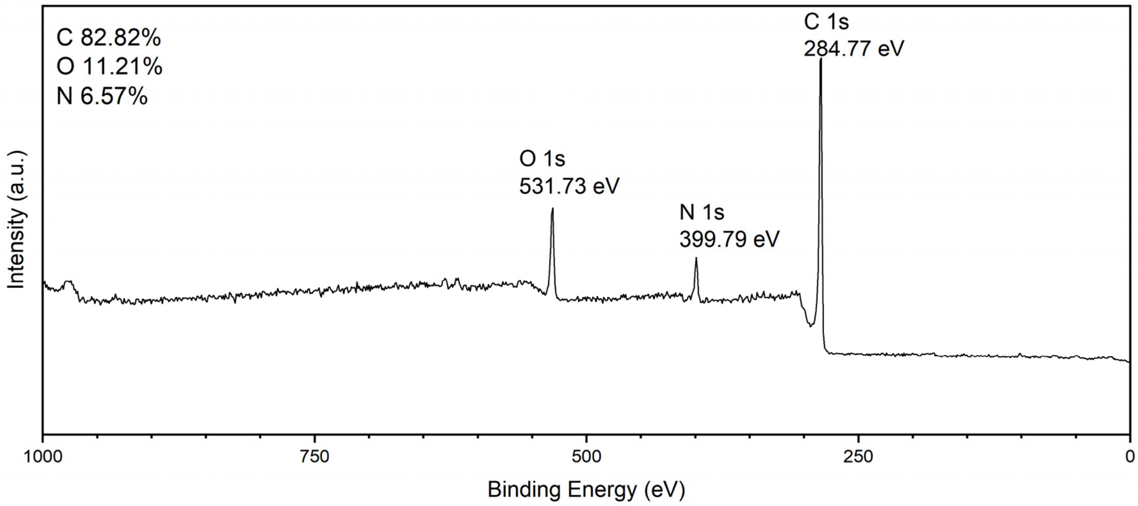

Figure S4. XPS total spectrum of HsGDY powder.

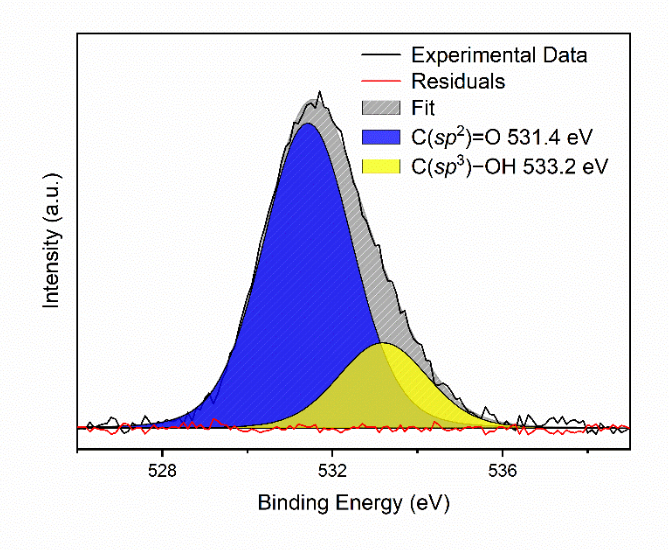

Figure S5. XPS high resolution of O 1s spectrum of HsGDY powder.

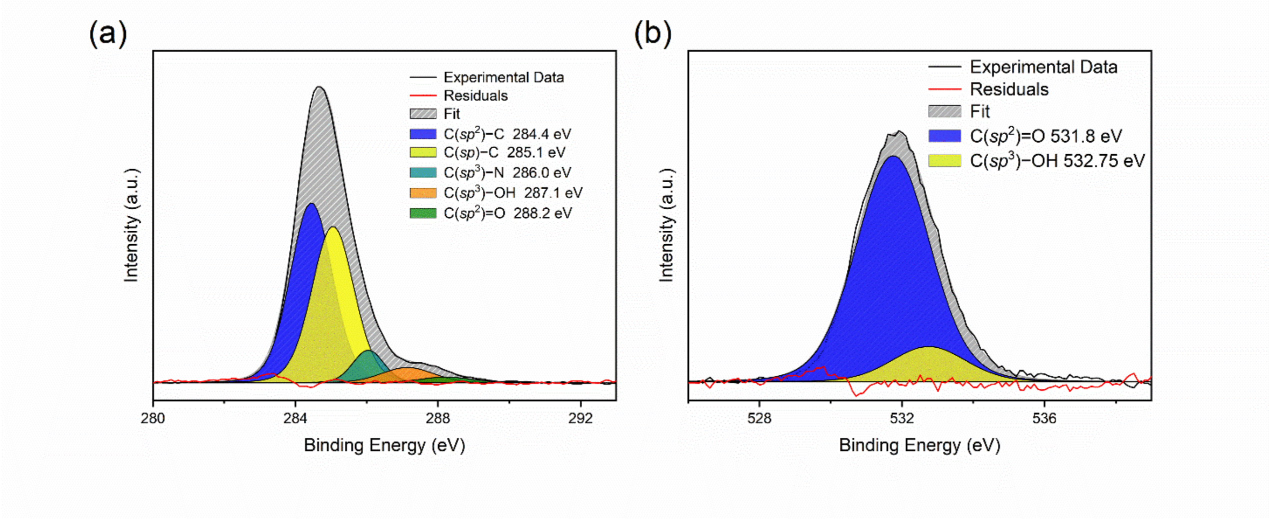

Figure S6. XPS high resolution of (a) C 1s and (b) O 1s spectrum of CPCM.

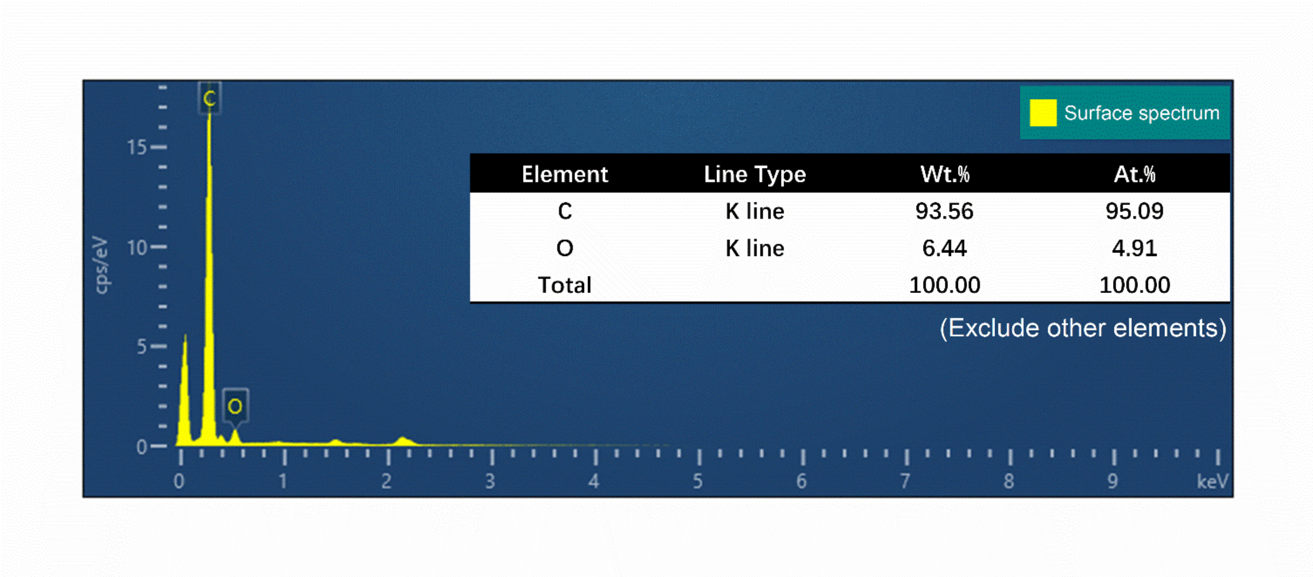

Figure S7. EDS analysis of HsGDY powder.

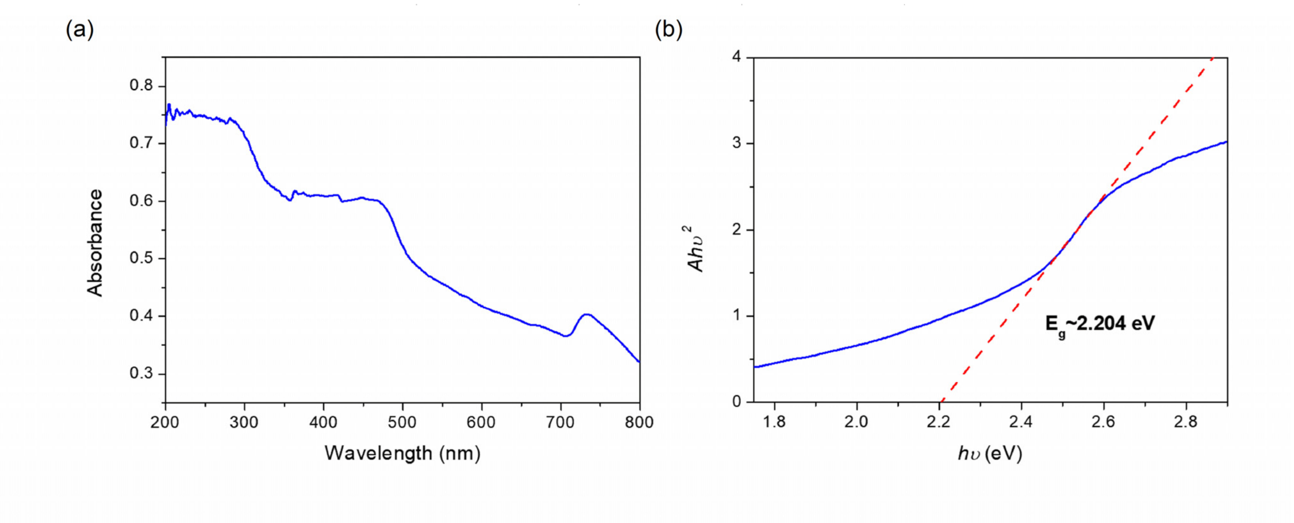

Figure S8. (a) The UV-visible spectrophotometry of HsGDY film (b) Determination of optical band gap of HsGDY film from the Tauc plot.
